# Influence of Social Media on the Dissemination and Uptake of Cardiology Research

**DOI:** 10.7759/cureus.86509

**Published:** 2025-06-21

**Authors:** Moeed Ali Karim

**Affiliations:** 1 Cardiology, University of Sydney, Sydney, AUS

**Keywords:** cardiology, research, social media, twitter, youtube

## Abstract

The digital revolution has transformed the landscape of scientific communication, offering new tools for the dissemination and uptake of research. In cardiology, a field defined by rapid innovation and large-scale data generation, social media platforms have emerged as powerful adjuncts to traditional publishing methods. Platforms such as X (formerly Twitter), YouTube, and LinkedIn are increasingly used by journals, academic institutions, and individual clinicians to share research, engage in discourse, and promote knowledge translation. This literature review explores the existing body of research on social media's role in the dissemination and uptake of cardiology research. It discusses the influence of altmetrics, the impact on citation patterns, the integration into conference proceedings and continuing medical education, and highlights ethical considerations, including misinformation and professionalism. The review concludes with future directions for research and policy in this emerging field.

## Introduction and background

Cardiovascular disease remains the leading cause of death globally, with continued research critical to informing prevention, diagnosis, and treatment efforts [1]. Over the past two decades, the volume of published cardiology research has grown substantially, driven by large-scale clinical trials, registries, and translational science [2]. Traditionally, peer-reviewed journals, conference presentations, and formal academic networks have served as the primary dissemination channels for new knowledge. However, these pathways can be limited by accessibility, time to publication, and geographical constraints.

Social media has emerged as a disruptive force in academic medicine. Defined as digital platforms that enable content creation and social interaction, social media facilitates real-time communication of research findings to broad and diverse audiences [3, 4]. A growing body of evidence suggests that platforms like Twitter, YouTube, and LinkedIn are now being used by cardiologists, researchers, professional societies, and journals to disseminate findings, stimulate discussion, and even influence clinical practice [5, 6].

This review synthesizes the existing literature on the influence of social media on the transmission and implementation of cardiology research. It explores how these platforms complement traditional dissemination models, evaluates the impact of online engagement on metrics of scholarly communication, and highlights risks and ethical concerns that must be addressed as the field evolves.

## Review

The rise of social media in cardiology

Over the past decade, social media has emerged as a transformative force in the field of cardiology, reshaping how clinicians, researchers, and institutions disseminate information, engage with peers, and interact with the public [7, 8]. The integration of platforms such as Twitter, Facebook, LinkedIn, and YouTube into professional practice has facilitated rapid communication, broadened the reach of scientific findings, and fostered a more interconnected global cardiology community.​

Adoption and Utilization Among Clinicians and Academics

The adoption of social media among healthcare professionals has seen a significant uptick. A study by Castro-Varela et al. (2020) highlighted that social media platforms are increasingly utilized by cardiovascular healthcare providers for academic communication, education, and networking [9]. These platforms enable clinicians to stay abreast of the latest research, participate in discussions, and share insights with a broader audience. The use of hashtags like #CardioTwitter has become commonplace, serving as a centralized hub for cardiology-related content, including journal club discussions, case studies, and conference highlights [10]. This digital aggregation facilitates real-time engagement and knowledge sharing across geographical boundaries.​

Institutional Engagement and Professional Societies

Professional societies have been at the forefront of integrating social media into their communication strategies. The American College of Cardiology (ACC) and the European Society of Cardiology (ESC) have developed robust social media presences to disseminate guidelines, research updates, and educational materials. For instance, the ACC's "Cardiology on the Go" initiative provides mobile-friendly content, including podcasts and video summaries, to cater to the evolving consumption habits of healthcare professionals [11]. Similarly, the ESC actively engages audiences during its annual congresses through live-tweeting sessions, interactive polls, and multimedia content, thereby enhancing accessibility and participant engagement [12]. These efforts not only amplify the reach of their content but also foster a sense of community among members.​

Impact on Research Dissemination and Academic Metrics

Social media has become an instrumental tool for researchers aiming to increase the visibility and impact of their work. Platforms like Twitter allow for the rapid dissemination of research findings, often accompanied by visual abstracts and infographics that distill complex information into digestible formats. This approach has been shown to enhance engagement and facilitate broader discussions [13, 14]. Moreover, the incorporation of alternative metrics, or "altmetrics," which track the attention an article receives online, has gained prominence. These metrics provide a more immediate measure of an article's reach and influence, complementing traditional citation-based metrics [15, 16]. Researchers increasingly include their social media handles and altmetric scores in curricula vitae and grant applications, recognizing the value of digital engagement in academic advancement.​

Role During the COVID-19 Pandemic

The COVID-19 pandemic underscored the critical role of social media in disseminating timely information. With the rapid evolution of knowledge regarding the virus and its cardiovascular implications, platforms like Twitter and LinkedIn became essential channels for sharing updates, guidelines, and research findings [17, 18]. A study by Ventola et al. (2014) emphasized that social media facilitates the swift exchange of information among healthcare professionals, enabling them to adapt to emerging evidence and best practices [19]. Additionally, they found that social media serves as a platform for virtual conferences and webinars, ensuring the continuity of professional development and collaboration during periods of physical distancing [20, 21]. The pandemic highlighted the adaptability and resilience of the cardiology community in leveraging digital tools to maintain the flow of information and support.​

Social media platforms and dissemination strategies

The advent of social media has revolutionized the dissemination of cardiology research, offering platforms that facilitate rapid sharing, engagement, and collaboration among professionals and the public alike. Various social media platforms serve distinct roles in this ecosystem, each contributing uniquely to the spread and impact of cardiovascular research.​

Twitter

Twitter has emerged as a central platform for academic cardiology, owing to its concise format and real-time communication capabilities. Its use has been associated with increased visibility and citation of research articles. A study by Luc et al. (2018) demonstrated that Twitter activity surrounding cardiovascular conferences correlated with higher article citations and abstract downloads [22]. The platform's ability to connect researchers, clinicians, and the public fosters a dynamic environment for knowledge exchange and discussion. ​

YouTube

YouTube serves as a valuable tool for in-depth educational content in cardiology. Institutions like the Mayo Clinic and the Cleveland Clinic maintain dedicated YouTube channels that provide video abstracts, surgical tutorials, and patient education materials [15]. These visual resources enhance understanding of complex procedures and concepts, making them accessible to a broader audience. The platform's widespread reach allows for the dissemination of information to both professionals and patients globally. ​

LinkedIn

LinkedIn offers a professional network where cardiologists and researchers can share updates, publications, and institutional news. While its reach may be more limited compared to other platforms, LinkedIn facilitates professional development and collaboration [16]. Organizations such as the ACC utilize LinkedIn to disseminate research findings and engage with the professional community. ​

Engagement Strategies in Social Media

Effective dissemination of cardiology research on social media involves strategic engagement practices [20]:​

Visual abstracts: Creating infographics that summarize research findings enhances comprehension and shareability. Visual abstracts have been shown to increase engagement and facilitate broader dissemination of research. ​

Twitter-based journal clubs: Initiatives like @CardioNerdsJC host discussions on recent publications, fostering community engagement and critical appraisal of research. These virtual journal clubs democratize access to scholarly discourse, allowing participation from a global audience. ​

Tagging and hashtags: Utilizing conference-specific hashtags (e.g., #ACC24, #ESCCongress) and tagging co-authors and institutions amplify the reach of posts, centralizing discussions and enhancing visibility. This practice facilitates networking and information sharing among professionals attending or following the events virtually. ​

These strategies not only increase the visibility of research but also foster a sense of community among geographically dispersed professionals, promoting collaboration and continuous learning [20].

Impact on research uptake

The integration of social media into academic cardiology has substantially influenced how research is disseminated, accessed, and utilized. Beyond traditional publication metrics, social media platforms offer alternative avenues for measuring and enhancing the reach and impact of scientific work. This section explores the role of alternative metrics (altmetrics) and the influence of social media on citation rates and clinical guideline dissemination [21].​

Altmetrics and Early Impact

Altmetrics, or alternative metrics, encompass a range of indicators that capture the digital footprint of scholarly work, including mentions on social media platforms, coverage in news outlets, policy document citations, and article downloads. Unlike traditional citation metrics, which may take years to accumulate, altmetrics provide a real-time snapshot of an article's reach and engagement within the broader community [15, 23].​

Eysenbach (2011) conducted a seminal study examining the relationship between Twitter activity and subsequent citation rates. The study found that articles tweeted within three days of publication were 11 times more likely to be highly cited later, suggesting that early social media attention can serve as a predictor of an article's future academic impact [24]. This finding underscores the potential of social media platforms to amplify the visibility of research shortly after publication. ​

The adoption of altmetrics has been facilitated by platforms like Altmetric.com, which integrate directly with many journal websites, allowing researchers to monitor the online attention their work receives. These metrics have become increasingly important for researchers seeking to demonstrate the broader impact of their work, particularly in grant applications and academic evaluations [25].​

Influence on Citations and Clinical Guidelines

While the correlation between social media exposure and increased citation rates does not necessarily imply causation, several studies have identified associations suggesting that social media promotion can enhance the academic impact of research. For instance, a randomized controlled trial by Luc et al. (2018) found that articles promoted on Twitter received significantly more citations after 12 months compared to non-promoted controls [22]. This study highlights the potential of social media strategies to augment the dissemination and uptake of scholarly work. ​

In the context of cardiology, social media has also played a pivotal role in the dissemination of clinical practice guidelines. Professional societies, such as the ESC, have effectively utilized social media platforms to share and summarize new guidelines. For example, the 2021 ESC Guidelines for Heart Failure were widely disseminated through social media channels, reaching an estimated 1.3 million accounts within the first month of publication [26]. Clinicians often shared infographics and concise summaries, facilitating the translation of complex recommendations into actionable insights for frontline providers.​

Moreover, social media platforms have enabled real-time discussions and debates surrounding new research findings and guidelines, fostering a more dynamic and interactive scientific community [27, 28]. This immediacy can accelerate the integration of new evidence into clinical practice, ultimately benefiting patient care.​

Integration into education and conferences

The integration of social media into cardiology has transformed medical education and the structure of professional conferences. Platforms such as Twitter, YouTube, and LinkedIn have become essential tools for disseminating knowledge, fostering interactive learning, and expanding the reach of academic events [23].​

Social Media in Medical Education

Social media platforms have emerged as innovative channels for medical education, offering accessible and interactive learning experiences [29, 30]. One notable example is the use of "tweetorials" on Twitter-threaded mini-lectures that provide concise, evidence-based teaching on cardiovascular topics, often supplemented with images or animations [31]. These tweetorials are widely utilized by both trainees and practicing clinicians to supplement traditional educational resources. An online survey conducted by Senapati et al. (2020) revealed that approximately 75% of respondents used social media as an educational tool, with Twitter being among the most preferred platforms for educational purposes [32].

The COVID-19 pandemic further accelerated the adoption of social media in medical education. With restrictions on in-person gatherings, educators and learners turned to digital platforms to continue educational activities. Virtual learning environments, including webinars, online journal clubs, and social media discussions, became commonplace [30, 33]. This shift not only ensured the continuity of education during the pandemic but also highlighted the potential of social media to enhance learning experiences beyond traditional classroom settings.

Social Media in Medical Conferences

Medical conferences have increasingly embraced social media to enhance engagement, disseminate information, and broaden their reach. Platforms like Twitter enable real-time commentary, allowing both attendees and those unable to attend to participate in discussions and access conference content. For instance, during the 2020 American Heart Association (AHA) Scientific Sessions, over 18,000 tweets were shared using the #AHA20 hashtag, resulting in more than 220 million impressions [34, 35]. ​

The use of official conference hashtags facilitates centralized discussions, making it easier for participants to follow specific topics and sessions. Additionally, social media allows for the sharing of visual abstracts, key takeaways, and expert opinions, enriching the conference experience. This digital engagement not only democratizes access to conference content but also fosters a sense of community among professionals across the globe.​ The pandemic also led to the emergence of entirely virtual conferences hosted through social media platforms [36, 37]. These virtual events, such as the International Cardio-Oncology Society Twitter Conference, offer accessible and cost-effective alternatives to traditional in-person gatherings. They provide opportunities for broader participation, especially for individuals who may face geographical or financial barriers to attending physical conferences.​

Challenges and Considerations

While the integration of social media into education and conferences offers numerous benefits, it also presents challenges. Ensuring the accuracy and credibility of information shared on social media is paramount [38, 39]. Educators and conference organizers must establish guidelines to maintain the quality and professionalism of content. Additionally, there is a need to address issues related to digital accessibility and to provide support for individuals less familiar with these platforms.​

Challenges

While social media has proven to be a powerful tool for disseminating cardiology research, it also introduces significant challenges. Prominent among these is the rapid spread of misinformation. In contrast to peer-reviewed journals, social media lacks a formal editorial process, which means that content shared on these platforms is not necessarily vetted for accuracy or context [40-42]. A content analysis by Chou et al. (2018) in JAMA revealed that approximately 20% of popular cardiovascular health tweets contained scientifically unsupported or inaccurate information. This finding highlights a troubling gap between engagement and credibility, especially when tweets originate from accounts with high follower counts, which can lend an unwarranted sense of legitimacy [43].

The speed and accessibility of social media are double-edged swords. While visual abstracts, tweetorials, and infographics simplify complex topics for broad audiences, they may inadvertently compromise the nuance of the original data. Research has shown that oversimplified content may lead to misinterpretation or inappropriate extrapolation of study results [44]. For instance, reducing a randomized controlled trial to a 280-character summary can obscure methodological limitations or subtleties in patient subgroups, thus altering clinical takeaways.

Furthermore, the informal nature of social media blurs the boundary between personal and professional communication. This raises ethical and legal concerns, particularly regarding patient confidentiality, data sharing, and potential conflicts of interest [19, 45, 46]. Clinicians are required to adhere to data protection standards, such as HIPAA in the United States and GDPR in Europe, even when using personal devices and platforms. Violations, intentional or otherwise, can damage professional reputations and compromise patient trust.

Recognizing these risks, several professional societies have issued guidance. The American Medical Association, the American College of Physicians, and the General Medical Council (UK) have published best practice guidelines that emphasize transparency, accurate referencing, avoidance of medical advice in public forums, and the clear separation of personal opinion from professional recommendations [19]. Training programs and institutions are increasingly offering workshops and curricula on ethical social media use, aiming to equip healthcare providers with the tools to engage responsibly online.

Democratization and equity

Despite these challenges, one of the most transformative aspects of social media in cardiology is its potential to democratize access to knowledge and professional networks. In traditional academic environments, dissemination of research often depends on institutional affiliations, journal subscriptions, and funding for conference attendance. Social media, however, breaks down many of these barriers, offering an open and largely cost-free platform for global participation.

Researchers in low- and middle-income countries (LMICs), for example, frequently face obstacles such as paywalled journals, limited research infrastructure, and restricted travel funding. Through platforms like Twitter and LinkedIn, they can now share their work, engage with international peers, and even participate in virtual conferences. A study by Tsao et al. (2021) noted a significant increase in social media-based collaborations and research visibility among LMIC investigators during the COVID-19 pandemic [17].

Early-career researchers and underrepresented groups also benefit from the leveling effect of social media. Initiatives such as #WIC (Women in Cardiology), #HeForShe, and #BlackInCardio have created digital spaces that promote equity, mentorship, and visibility for historically marginalized groups in cardiovascular medicine. These hashtags not only showcase achievements and academic milestones but also serve as platforms for discussing systemic barriers within the field.

Importantly, patients and advocacy organizations are also leveraging social media to influence cardiovascular research. Groups such as the Global Heart Hub and Mended Hearts use Twitter and Facebook to highlight patient priorities, promote awareness campaigns, and disseminate patient-centered guidelines. This grassroots engagement allows for more inclusive and responsive research agendas, particularly in areas like heart failure, congenital heart disease, and cardiomyopathies.

Future directions

More robust research into the causal relationship between social media activity and traditional academic outcomes remains needed. Larger, multi-institutional trials should assess whether specific social media strategies improve practice adoption, patient outcomes, or knowledge retention. Moreover, institutions should provide formal training in digital literacy and content creation. Medical schools and postgraduate programs could integrate social media into communication curricula, addressing both opportunities and pitfalls. Artificial intelligence tools may eventually be deployed to summarize articles, detect misinformation, and tailor content to user needs. As platforms evolve, cardiologists will need to stay agile, adapting their communication strategies to meet emerging formats such as podcasts, TikTok, or the Metaverse [46].

## Conclusions

Social media is no longer a peripheral tool in cardiology, it is a core component of research dissemination, professional engagement, and clinical education. While it cannot and should not replace traditional peer-reviewed publication, it offers powerful complementary advantages: rapid visibility, global reach, and interactive learning. The cardiology community must embrace these platforms while remaining vigilant about ethics, accuracy, and equity. With thoughtful integration, social media can accelerate the translation of research into practice and ultimately improve cardiovascular outcomes worldwide.
